# A Geoprivacy by Design Guideline for Research Campaigns That Use Participatory Sensing Data

**DOI:** 10.1177/1556264618759877

**Published:** 2018-04-23

**Authors:** Ourania Kounadi, Bernd Resch

**Affiliations:** 1 University of Salzburg, Austria; 2 Center for Geographic Analysis, Harvard University, Cambridge, MA, USA

**Keywords:** geoprivacy by design, location privacy, spatiotemporal data, mobile participatory sensors, disclosure risk, anonymization methods, research design, spatial analysis

## Abstract

Participatory sensing applications collect personal data of monitored subjects along with their spatial or spatiotemporal stamps. The attributes of a monitored subject can be private, sensitive, or confidential information. Also, the spatial or spatiotemporal attributes are prone to inferential disclosure of private information. Although there is extensive problem-oriented literature on geoinformation disclosure, our work provides a clear guideline with practical relevance, containing the steps that a research campaign should follow to preserve the participants’ privacy. We first examine the technical aspects of geoprivacy in the context of participatory sensing data. Then, we propose privacy-preserving steps in four categories, namely, ensuring secure and safe settings, actions prior to the start of a research survey, processing and analysis of collected data, and safe disclosure of datasets and research deliverables.

## Introduction

Participatory sensing refers to sensor data gained voluntarily from participants for personal benefits or to benefit the community (Christin, Reinhardt, Kanhere, & Hollick, 2011). Sensors are attached to mobile devices such as smartphones or smart wristbands, and typically collect data to be examined (e.g., heart rate) along with other sensed data such as location, time, pictures, sound, and video. The main sensing measurement can be collected for personal interest such as the *BALANCE* system that detects the caloric expenditure of a user (Denning et al., 2009). Another application of participatory sensing is to alert medical staff of their patients’ abnormal behaviors like the *MobAsthma* application that measures asthma peak flows, pollution, and location to inform on asthma attacks (Kanjo, Bacon, Roberts, & Landshoff, 2009). These applications are human centric because they collect information about the individual who carries the sensor. There are also environment-centric applications, where the participant acts as a “human as sensor operator” and carries the mobile device to capture environmental phenomena such as air quality or noise (Kanjo et al., 2009; Maisonneuve, Stevens, Niessen, & Steels, 2009).

Also, participatory sensing has been used for spatial as well as a-spatial research studies. The *EmbaGIS* application depicts stress-level peaks in the movement of handicapped people for the identification of urban barriers (Rodrigues da Silva, Zeile, de Oliveira Aguiar, Papastefanou, & Bergner, 2014). An a-spatial example is the *HealthSense* project that improves the classification of health detection events through user feedback information incorporated into machine learning techniques (Stuntebeck, Davis, Abowd, & Blount, 2008). The application examples mentioned so far collect and analyze objective measurements from sensors. However, in some spatial studies subjective measurements (i.e., provided by the participant via a questionnaire app) are collected to either complement objective measurements of biometric sensors (Resch, Summa, Sagl, Zeile, & Exner, 2015), or measure emotions and perceptions (e.g., fear of crime, happiness, perception of environmental and built phenomena, or mood) that are more difficult to capture via biometric sensors (MacKerron & Mourato, 2013; Solymosi, Bowers, & Fujiyama, 2015; Törnros et al., 2016; Zeile, Memmel, & Exner, 2012).

The usage of spatiotemporal participatory sensing data is a scientific trend in many fields, and the intensity of the studies is expected to increase in the future. However, these data entail significant privacy violations risks, partially due to their complexity, and partially because practitioners and the public are not fully aware of the potential disclosure risks linked to these data. With respect to the usage of participatory sensing data in research studies, Resch (2013) denotes the practitioners’ obligation to address several privacy issues such as data ownership, accessibility, integrity, liability, and participants’ opt-in/opt-out possibility. However, practitioners are not always aware of privacy implications, methods for protection, and how and when to apply them in research. Three studies in the fields of medicine, health geography, sexual and reproductive health, GIScience, geography, and spatial crime analysis examined how confidential point data of participants were portrayed on maps, and found numerous cases where original data were used instead of aggregated or anonymized data (Brownstein, Cassa, & Mandl, 2006b; Haley et al., 2016; Kounadi & Leitner, 2014). The studies cover a period between 1994 and 2015, and their findings remain consistent; efforts to instill sensitivity to location privacy and disclosure risk have been relatively unsuccessful, and researchers ignore or are unaware of the spatial reidentification risk when publishing point data on maps. The findings reveal the need for educating practitioners over privacy and confidentiality issues with the use of spatial data.

Our article aims to establish a general guidelines framework for privacy-preserving tasks during a research campaign that collects participatory sensing data. The term “research campaign” encompasses two possible research efforts: First, an institution or research group not only conducts surveys for their studies, but they may also consider to publish the data, share them with other members of the institution or with third parties. Second, a research group or an individual researcher collects survey data for a single study. In the next sections, we analyze privacy issues and practices (sections “Geoprivacy, Confidentiality, and Spatial Datasets” and “Essential Technical Analysis”), and then propose recommendations for the different stages of a research campaign (section “Privacy by Design Research Campaign”).

## Geoprivacy, Confidentiality, and Spatial Datasets

Although privacy has been conceptualized and explored for quite sometime (Post, 2001; Waldo, Herbert, & Lin Millett, 2007; Westin, 1968), privacy regarding spatial data is described with separate definitions and in sometimes distinguished by the type of spatial dataset that it addresses. A general definition that describes well geoprivacy for both confidential discrete location data and spatiotemporal trajectories of individuals by Kwan, Casas, and Schmitz (2004) denotes that geoprivacy refers to

individual rights to prevent disclosure of the location of one’s home, workplace, daily activities, or trips. The purpose of protecting geo-privacy is to prevent individuals from being identified through locational information (p. 3).

The disclosure of locations may compromise individual privacy when these are used to infer personal information about an individual (e.g., living place, working place, frequently visited places). In addition, confidentiality can be breached if the disclosed locations are linked to one or more sensitive attributes such as in confidential discrete location datasets. Thus, spatial datasets may pose risks to both the privacy and confidentiality of the entities.

Regarding participatory sensing data, Christin et al. (2011) provided a definition that gives full control of the disclosed information to the users of a participatory sensing application:

Privacy in participatory sensing is the guarantee that participants maintain control over the release of their sensitive information. This includes the protection of information that can be inferred from both the sensor readings themselves as well as from the interaction of the users with the participatory sensing system (p. 1934).

The definition above describes privacy with respect to e-diaries, health monitoring, or other applications. However, when it comes to data that need to be collected for research purposes the disclosed information should be predefined in a confidentiality–participation agreement, and thus the control is transferred to the trusted data holders (i.e., controller).

Overall, geoprivacy definitions do not encompass all types and applications of spatial data that are prone to compromising individual privacy and/or confidentiality. For certain types, such as the collection of data through a survey, a *spatial confidentiality* definition would be more appropriate to use than a location privacy definition. The complexity and several dimensions of the confidentiality and privacy risks linked to spatial data make the formulation of a single definition extremely difficult, if not impossible. However, there exist anonymization methods that have not only been developed for one datatype but can also be applied to another. Furthermore, some privacy threats that were mentioned for one datatype may have been neglected or unacknowledged for another datatype that has similar risk of reidentification. This shows that privacy and confidentiality literature for location data has to be examined more broadly to bring complete solutions. The spatial data that are at risk of disclosing private or confidential information are listed below. Our categorization is subjective and aims at highlighting the differences of the categories that they have an effect on the geoprivacy strategy to be implemented:

1. Mobile phone data
2. Location-based services (LBS) data
3. Location-based social network (LBSN) data
4. Confidential discrete location data
5. Confidential discrete location data on individuals
6. Sensitive discrete location data on individuals
7. Data from mobile technical sensors carried by “humans as sensor operators”
8. Data from mobile technical sensors carried by “humans as objective sensors”
9. Data from mobile devices carried by “humans as subjective sensors”

Mobile phone data contain the users’ past locations attached with their time stamp and other phone-related attributes depending on the dataset. The spatiotemporal accuracy may vary depending on the population density, the method of extracting locations, and the type of dataset. Typically, in areas with high population density, such as cities and towns, the spatiotemporal accuracy is high. A typical example of the second type are applications for navigation services that, like the first type, may collect spatial and temporal information of their users. In the third dataset, a user has the option to disclose his or her location along with the time stamp and the attribute information that is inherent in most social media applications (e.g., a text on Twitter). The fourth location dataset is the least discussed in the literature of location privacy. An exemplary dataset here is the Incident and Trafficking Database (ITDB) by the International Atomic Energy Agency enclosing the illegal movement of nuclear and radioactive materials (International Atomic Energy Agency, 2015). The fifth and sixth datatypes have been mostly discussed for health and crime geocoded datasets such as the residential locations of patients of a disease or household locations of victims of a crime. The next three datatypes refer to spatiotemporal data collected from participatory mobile sensing applications. The “human as sensor operators” refers to examples where users of mobile phones capture environmentally related information such as noise, traffic, and air quality. However, to project this information spatially the temporal and spatial information of the users is captured as well. The eighth datatype involves physiological measurements of the individual who carries the device such as data from biometric sensors used for health-monitoring purposes. In the last type, the data subjects act as sensors similar to Datatype 8, but they report their own subjective perceptions of the sensed attribute, which can be either about the environment (e.g., public safety, quality of life, or road safety) or about themselves (e.g., fear or emotions). This is typically done with a smartphone application that sends requests to the participants to enter their emotions or perceptions instantly, or at their earliest convenience (based on experience sampling method).

Each of the nine datasets has certain characteristics due to which protection approaches may differ between categories of data. A LBS dataset may not only have similar attributes to a mobile phone dataset, but it may also have significant differences in its temporal frequency. The text attributes of a LBSN dataset may lead to inferential disclosure of personal preferences, opinions, and other private matters. The fourth dataset is about confidential locations (e.g., a location where a radioactive material was stolen), and the fifth dataset is about confidential location data on individuals (e.g., the home location of a patient who has been diagnosed with a certain disease). The approaches to protect the abovementioned datasets (i.e., method, anonymity measure, anonymity level as requested by authorities and institutions, and data to assess the disclosure risk) shall be different.

Furthermore, Datatypes 8 and 9 can be considered as the most complex ones due to the variety and sensitivity of personal information that is collected (i.e., spatial, temporal, and sensitive/confidential). Also, for research purposes additional attributes of the data subjects and/or a combination of subjective and objective measurements can be collected. Our recommendations focus on Datatypes 8 and 9 because their complexity and sensitivity can lead to greater privacy loss compared with the other datasets.

## Essential Technical Analysis

### Disclosure Risk of Released Data and Deliverables

The comprehension of disclosure risk and reidentification techniques is critical to design efficient privacy implementations. Below, we present a list of release scenarios for research efforts that collect microdata and associated deliverables of Datatypes 8 and 9. Each scenario is analyzed in terms of the risk of disclosure and privacy threats to the data subjects. The location protection methods and research guidelines in the next sections take into consideration these scenarios. However, we do not claim that this is an exhaustive list.

- Scenario 1: Disclosure of original data

The full dataset is disclosed that includes the values for each objective or subjective measurement (or both), the spatial and temporal stamps, as well as the identity of the measurement’s subject.

Data from Scenario 1 are prone to similar inference attacks to data collected in LBSNs. According to Alrayes and Abdelmoty (2014), LBSNs contain three types of semantics: the spatial semantics that can be used to infer places visited, the nonspatial semantics which are mostly textual information for LBSN, whereas for participatory sensing these semantics are the subjective or objective measurements, and the temporal semantics revealing the time and duration of a visited place. We filtered out privacy threats from inference attacks that were discussed by the aforementioned authors based on their common characteristics with participatory sensing data. The following personal information can be inferred: (a) home location, (b) work location, (c) most visited places and time spent at these places, (d) locations and activities during weekends, (e) lunch places and after-work activities, (f) favorite stores, (g) time spent away from home, and (h) time spent away from work. In addition to these eight privacy threats, the participants of the study will be known, and sensitive private information depending on the measurement will be revealed. This extreme scenario leads to a far-reaching loss of privacy and involves all types of disclosures (i.e., identity, attribute, and inferential—for definitions, refer to the supporting information file). It is also worth mentioning other serious privacy threats that have been identified related to the use of mobile sensing applications such as identity theft, profiling, stalking, embarrassment, extortion, and cooperate use/misuse (Barcena, Wueest, & Lau, 2014).

- Scenario 2: Disclosure of key identifiers

A dataset is disclosed that includes the values for each objective or subjective measurement (or both), the spatial and temporal stamps, as well as one or more key identifiers of the measurement’s subject.

While a full name is not present in the dataset, other identifying elements may be given such as e-mail or home address. E-mail addresses can be linked with other online sources to reveal the identity of a participant. Furthermore, home addresses can disclose the participants’ identities, especially in purely residential single family areas (i.e., a location depicts a residence of only one household). Even if the home address is given as a set of geographical coordinates, *X* and *Y*, instead of textual information, the latter can be inferred using freely available reverse geocoding services (Kounadi, Lampoltshammer, Leitner, & Heistracher, 2013).

- Scenario 3: Disclosure of pseudonyms

A dataset is disclosed that includes the values for each objective or subjective measurement (or both), the spatial and temporal stamps, as well as a pseudonym representing the measurement’s subject.

This scenario illustrates the inferential disclosure of such datasets with the use of data mining and geoprocessing techniques. If a participant is distinguished by an id, a subset of location data can be analyzed to infer his or her home address that will lead to privacy threats mentioned in Scenario 1. The space–time stamps of a participant can be translated to trips with distinguishable start and ending destinations. What if the ending destination of a participant for trips after 10:00 p.m. is frequently on the same or a nearby location? This location can be the participant’s home location. Krumm (2007) analyzed subjects’ trips for a recording period of a minimum 2 weeks and tried to infer their home locations using several algorithms. The median distance error of the real home address to the inferred one was 60.7 m. Similar approaches may be used for most inference attacks mentioned in Scenario 1. The spatial reidentification risk of data from participatory sensing applications depends on the recording period, the residential patchiness study area, and the frequency of the space–time stamps. Although specific reidentification studies for participatory sensing data do not exist, previous findings from other spatial datatypes pinpoint the risk that should not be neglected.

- Scenario 4: Disclosure of quasi-identifiers and data collection meta-data

A dataset is disclosed that includes the values for each objective or subjective measurement (or both), the spatial and temporal stamps, as well as one or more quasi identifiers of the measurement’s subject.

Identity or attribute disclosure is difficult to achieve when quasi-identifiers (e.g., socioeconomic characteristics of a subject) exist in a dataset that has multiple and variable measurements per participant. This is because a subset of measurements cannot be linked to an individual. However, if there are only a couple of measurements with the same combination of quasi-identifiers it can be inferred that they belong to a single individual. Also, if the controller discloses information on the data collection methods (e.g., there are a minimum or predefined number of measurements per participant), this information can be used to define a subset of measurements for one or more data subjects. For example, a study collects 100 measurements per participant, and discloses this dataset along with the sex and the occupation of each measurement’s subject. A subsequent data analysis filters out 100 measurements of a man of occupation “X.” All measurements refer to one individual, which is known due to data collection meta-data information. Also, it can be found that there is only one man of this occupation in the study area. Thus, the identity and attribute disclosure of this participant have been compromised like Scenarios 1 and 2.

- Scenario 5: Identifying participants in a digital map or printed map

A map is disclosed in a digital or printed format that portrays the locations and/or values of the measurements for one or more participants.

Data deliverables such as participants’ maps are also prone to reidentification. For example, a map is uploaded on a website of a research organization portraying the values and locations of the measurements for one participant. Reengineering can be applied to the point map to extract the geographical coordinates of the participant’s locations. Brownstein et al. (2006a) applied a reengineering process that involves an unsupervised classification to examine the spatial reidentification risk of the publication of high- and low-resolution point maps. The number of correctly reengineered addresses was 79% for the high-resolution map and 26% for the low-resolution map, indicating that by lowering the resolution of a digital map does not prevent reidentification. Once the coordinates of the participant are extracted, the home address can be estimated (Scenario 3), then reverse identification (Scenario 2) will reveal a single address or a set of addresses, and finally addresses can be used to infer the identity of the participant. The disclosure remains even if the map is in a printed format. In this case, the map can be scanned and georeferenced to a known coordinate system. The reengineering error of a point printed map was examined by Leitner, Mills, and Curtis (2007) who found that the distance errors (i.e., distance from the actual to the reengineered location) ranged from 59.54 m to 156.63 m, and are independent of the map scale.

- Scenario 6: Multiple versions of anonymized datasets

The controller releases multiple versions of anonymised copies of the original data.

In this scenario, original data are first anonymized using an anonymization method. The controller shares the anonymized data with a research firm, and soon after discards them because he or she owns the original data. After sometime, another research firm may make a request for an anonymized copy. The controller reapplies the anonymization method that incorporates a randomization function, and therefore the anonymized copy is different than the first one. The more this process is repeated, the more copies are distributed that increase the spatial reidentification risk of the original data. Multiple versions of anonymized dataset may give hints regarding the method’s parameters and characteristics to an attacker who will try to reidentify the original data. This scenario has been tested and confirmed for the “non-deterministic Gaussian skew” location protection method (Cassa, Wieland, & Mandl, 2008).

- Scenario 7: Disclosure of anonymization meta-data

The controller releases metadata information on the location protection method and/or additional disclosure limitation practices applied to the original data.

Controllers often disclose meta-data regarding the location protection method or any other disclosure limitation technique that is applied to the original data to ensure that confidentiality and privacy of subjects are protected, and also to provide information on the spatial information loss of the anonymized released copy that may be used and analyzed by others. However, reengineering can be improved with the disclosure of anonymization meta-data because, just like Scenario 6, it provides hints to a potential attacker. This has been tested with methods such as aggregation and perturbation (Zimmerman & Pavlik, 2008).

### Disclosure Risk of Data Collection and Storing on Devices

*Data security* has been characterized by Boulos, Curtis, and AbdelMalik (2009) as the “missing ring” in privacy-preserving discussions. The authors describe a scenario of a research study that has a well-defined privacy-preserving plan, has been approved by an institutional review board (IRB), and employs adequate practices for the publication of results and maps. However, the security components are not checked and approved as the other parts of the research study such as the subjects’ consent to conduct the study, disclosure risk of analysis, reporting findings, and sharing data. Thus, the research process is likely to neglect risks regarding data theft, data loss, or data disclosure to nonauthorized parties.

Tracking devices that collect physiological or subjective measurements can be smartphone applications that collect responses to emotions and perceptions, smartphone applications that exploit built-in sensors, or wearable tracking devices such as a wristband or a watch. The measurements are stored in databases locally, remotely, or both. Data are viewed and analyzed via computer (smartphone, desktop, or laptop), and frequently require Internet access (i.e., cloud-based model). Based on the structure of self-tracking systems, security risks exist when data are stored on the device, data are stored in the cloud, and data are transmitted to the cloud. Barcena et al. (2014) examined a range of self-tracking services regarding the security issues that take place during the storing or transmission of data. First, they found that Bluetooth-Low-Energy-enabled devices can transmit a signal that can be read by scanning devices and provide an estimate location of the device. Therefore, the spatiotemporal patterns of the users can be leaked (the same applies when Wi-Fi is enabled on the device). Second, 20% of the examined applications that offer cloud-based service components may transmit login credentials in clear text (i.e., nonencrypted data). Third, the examined services contacted on an average five unique domains. These domains receive information on the user’s behavior and activities without the users being aware of it. Fourth, the services employ user account-based services that make the sessions insecure and potential to be hijacked. Fifth, data leakage may occur if applications use third-party services. Last but not least, half of the existing services do not have or do not make available their privacy policies.

Several security and anonymity frameworks, however, have been proposed for participatory sensing applications (De Cristofaro & Soriente, 2011; Shin et al., 2011; X. O. Wang, Cheng, Mohapatra, & Abdelzaher, 2013). These frameworks provide mechanisms to preserve users’ privacy when their data are reported in the cloud to a service provider. However, we should outline here that in the context of a research campaign it is not necessary to send and store data in the cloud or to involve a third-party service provider.

### Anonymization Methods

In this section, we refer to widely discussed anonymization methods (Table 1) that aim to protect from Disclosure Scenarios 1 to 5. However, we should outline that most of the methods have not been evaluated for Scenarios 6 and 7 on meta-data disclosure or multiple versions of anonymized copies. The methods mostly affect the precision or the accuracy of the produced anonymized (“masked”) data. Precision refers to the exactness of information (in geographical terms, it is the number of decimal places of the latitude and longitude of locations), whereas accuracy is the relation between a measured value and the ground truth. In general, “precision-affecting” methods are accurate with respect to the information they report, and “accuracy-affecting” methods are fairly precise. For example, if an observation is aggregated into a postcode level it is not as precise as a point-level observation, but the information that the observation lies within the postcode is accurate. Similarly, if an observation is translated 300 m to the north it is very precise but still inaccurate.

**Table 1.** Privacy and Confidentiality Approaches for Statistical and Spatial Data.

Dataset	Anonymization approaches	Description	Major effect	Benefits	Limitations
Microdata	Abbreviation	Reduces the volume or granularity of released information	Imprecision	Easy implementation; mathematical basis for location protection methods	Current applications are restricted to a-spatial data
	Aggregation	Combines adjacent categories or replaces with nearby values			
	Modification	Changes data values with rounding or perturbation	Inaccuracy		
	Fabrication	Creates a fictional dataset that has distributional and inferential similarities with the original			
Confidential discrete spatial data (e.g., health care, crime, household surveys)	Adaptive geomasking	Actual locations are perturbed considering the spatial k-anonymity	Inaccuracy	Risk of identification information can be adaptively anonymized to meet data-specific regulations and restrictions; anonymized data retain the initial discrete structure that is crucial for many spatial-point pattern analyses	Current applications are restricted to static, nontemporal discrete location data
	Geomasking with quasi-identifiers	Geographical masks that extend spatial k-anonymity to basic k-anonymity to account for quasi-identifiers	Inaccuracy or imprecision	In addition to the location and sensitive theme, quasi-identifiers may be disclosed that allow further analysis of covariates	
	Synthetic geographies	Anonymized data are synthesized from the results of spatial estimation models that use covariates as estimators of confidential locations	Inaccuracy	Retains relationship between locations and covariates	
Spatiotemporal data of individuals (e.g., GPS trajectories, cellular data, LBS, radio-frequency identification devices [RFID])	Point aggregation	A set of locations is replaced by a single representative location	Imprecision	Adequate for visualizing trajectories of individuals or movement flows in between areas	Point aggregation underperforms random perturbation techniques
	Cloaking	Lowers the space and/or time precision of individual-level data		Option to decrease the temporal or the spatial resolution	Prohibits spatial-point pattern analysis; polygon clustering may hide significant point clusters
	Dummies	Adds noise that simulates human trajectories	Inaccuracy	Allows spatial-point pattern analysis and analysis by user	The spatial accuracy of the augmented anonymized dataset compared with the original one has not been addressed
	Pseudonyms	Identities are stored with pseudonyms			Inferential disclosure is not protected
	Mix zones	Locations are hidden in certain areas, and pseudonyms change when exiting them		High positional accuracy is achieved in low sensitivity areas; it is harder, if not impossible, to perform inference attacks on individuals’ spatiotemporal behavior if pseudonyms are changed periodically	Analysis by user or group of users is not possible if pseudonyms change in time

*Note.* GPS = global positioning system; LBS = location-based services.

Early methods are mainly statistical and were developed for the protection of microdata. Due to the nature of the data, the methods are applied to a matrix in which each row is a subject and each column an attribute. Although the structure of participatory sensing spatiotemporal data is different, these methods formed the basis for the next generation of more advanced techniques, including the spatial or the spatiotemporal ones. They can be summarized into four categories: abbreviation, aggregation, modification, and fabrication (Cox, 1996). An example of abbreviation is the suppression of records (in this context, it means removal) from geographical areas of low population density. In aggregation, microdata records (one record equals to one data subject) of similar values can be averaged, and therefore microdata are transformed to tabular data. A typical example of modification is perturbation where random noise is added to each cell or to certain variables. Last, one fabrication technique is data swapping between records in a way that predefined cross-tabulations are preserved. Also, most techniques can be applied to the records of the matrix (i.e., record transforming masks) or to the columns of the matrix (i.e., attribute transforming mask; Duncan & Pearson, 1991).

The first generation of anonymization methods for confidential discrete spatial datasets, commonly known as “geomasking-techniques,” is based on existing methods on microdata such as aggregation and modification with specific adaptations to protect the spatial attribute of the data. According to Zandbergen (2014), “Geographic masking is the process of altering the coordinates of point location data to limit the risk of re-identification upon release of the data (p. 4).” The alteration of the coordinates produces an aggregated dataset or a modified dataset depending on the technique to be used. If points are aggregated into areal units, the transformed dataset has fewer entities than the original dataset with count data for each one of them, similar to microdata aggregation. If points are aggregated into a new set of symbolic or surrogate points, the transformed dataset may retain the original number of observations (Armstrong, Rushton, & Zimmerman, 1999; Leitner & Curtis, 2004). Regarding the modification of the coordinates, points can be processed at a global level with an affine transformation (Armstrong et al., 1999) or other cartographic techniques such as flipping and rotation (Leitner & Curtis, 2004), and at a local level by modifying points with approaches based on random perturbation (Kwan et al., 2004; Leitner & Curtis, 2004), or snapping them along the edges of their corresponding Voronoi polygon (Seidl, Paulus, Jankowski, & Regenfelder, 2015).

Adaptive geomasking techniques are modification techniques that displace original point locations within uncertainty areas, where the sizes of these areas are defined by the underlying population density. The purpose of these techniques is to offer a “spatial k-anonymity,” meaning that each confidential or private location on the dataset (e.g., a household) cannot be distinguished among k-1 other locations. Spatial k-anonymity is an adaptation of the classic k-anonymity model. K-anonymity ensures that an effort to identify information of an entity ambiguously maps information to at least k entities; in other words, any group is hidden in a group of size k regarding the quasi-identifiers (Samarati & Sweeney, 1998). The uncertainty area of the “population-density-based Gaussian spatial blurring” is circular, and the selection of the displacement is based on a normal distribution (Cassa, Grannis, Overhage, & Mandl, 2006). In “donut geomasking,” the uncertainty area has the form of a torus so as to ensure a minimal displacement (Hampton et al., 2010).

Furthermore, the “voronoi-based aggregation system” (Croft, Shi, Sack, & Corriveau, 2016; a spatial aggregation approach) and the “triangular displacement” (a modification approach; Murad, Hilton, Horan, & Tangenberg, 2014) can be applied to spatial datasets that include covariates, although there are still open questions with respect to the spatial analytical error they produce (regarding the Voronoi-based method) or the quantification of the offered k-anonymity (regarding the triangular displacement method). Last, concepts of simulated geographies (a fabrication approach) also require additional attributes to create a protected spatial dataset (Paiva, Chakraborty, Reiter, & Gelfand, 2014; H. Wang & Reiter, 2012). Here, the attributes are used to make spatial predictions on the confidential theme. The resulting hotspots are then used to synthesize the anonymized dataset.

The general drawback of techniques on confidential discrete spatial data is that they have not been applied to spatiotemporal data. Tuning of the algorithms is needed to consider multiple sensitive measurements per data subject as opposed to traditional confidential discrete data where one location, typically a home address, is given per subject. However, an important advantage of geomasking studies for privacy research design is the extensive evaluation of the produced masked datasets regarding the spatial analytical error.

Spatial-point aggregation (Adrienko & Adrienko, 2011; Monreale et al., 2010), or spatial-areal and temporal aggregation, known also as cloaking (Cheng, Zhang, Bertino, & Prabhakar, 2006; Gruteser & Grunwald, 2003; Kalnis, Ghinita, Mouratidis, & Papadias, 2007), follows the same approach as statistical aggregation. In particular, it decreases the precision of original data. Point aggregation can be used for both privacy protection and a generalization approach to visualize flows in movements and in between areas. With cloaking, the time duration of an object at one location is considered as quasi-identifier. Given the number of other objects at this location and for this time duration, a decision to decrease spatial resolution will be taken. Similarly, one can lower the temporal resolution. Because cloaking is designed for LBS data, the anonymity it offers is calculated based on the number of other data subjects (i.e., users of a service) at a particular time and location. Considering the number of users of a LBS, this approach can provide sufficient anonymity. However, the number of participants in participatory sensing studies will probably be much lower, and this will greatly affect the anonymized dataset’s spatial precision due to larger disclosed regions and/or coarser time. Generally, all techniques that involve some sort of spatial aggregation will affect analytical results due to the modifiable areal unit problem (Openshaw & Openshaw, 1984). In practice, polygon or point clusters of the measurements’ values may appear or disappear depending on the aggregation’s division of the space.

A different concept is to add noise to the data with artificial trajectories so called “dummies”(Kido, Yanagisawa, & Satoh, 2005; You, Peng, & Lee, 2007). Dummies are added to satisfy the anonymity of each data subject. Although dummies are an interesting approach, the spatial analytical errors of the increased dataset have not been addressed and should be considered when such a dataset is released for research purposes. Another technique that affects the accuracy of the data is the use of “unlinked pseudonyms” that are fake identities associated with data subjects (Cuellar, 2004). As it is explained earlier, pseudonyms will not prevent inferential disclosure when space–time stamps are disclosed. A more sophisticated version of pseudonyms is the “mix zones” method in which a new pseudonym is given to a subject as soon as he or she exits the so called mix zone (Beresford & Stajano, 2003, 2004; Buttyán, Holczer, & Vajda, 2007). In addition, while being in the mix zone locations are hidden. There are two limitations to be considered if such methods are to be exploited for participatory sensing data: First, they take into consideration only the space and time attributes, whereas participatory sensing data also include confidential measurements and potentially additional quasi-identifiers. Second, the anonymity refers to other or artificially inserted subjects in the dataset (i.e., users of a service), which may not prevent disclosure of private locations (see Scenario 3), unless either the underlying residential/building structure is considered or a very large number of participations in the study are achieved.

The presented methods have the potential to be used for participatory sensing data if they are combined and/or adapted. Nevertheless, the complexity of a participatory sensing dataset has to be taken into account. Specifically, a spatiotemporal trajectory dataset contains the attributes for each data subject for multiple measurements per subject, like a participatory sensing dataset. However, it does not have sensitive attributes or quasi-identifiers other than the spatiotemporal information. On the contrary, a confidential discrete dataset may have quasi-identifiers and sensitive attributes but collects only a single measurement for each data subject.

Another limitation of the existing techniques is that most of them are based on the concepts of spatial k-anonymity and k-anonymity aiming at decreasing the risk of inferential disclosure or identity disclosure. These concepts cannot prevent attribute disclosure that may occur from homogeneity attack (i.e., knowing a person who is in the database) and background knowledge attack (i.e., knowing a person who is in the database, and additional information on the distribution of the sensitive attribute or on the characteristics of the person who is in a database). The problems can be solved with the concept of “l-diversity” where an equivalent class has at least l “well-represented” values for the sensitive attributes (Machanavajjhala, Kifer, Gehrke, & Venkitasubramaniam, 2007). L-diversity ensures that for one sensitive attribute table, all equivalent classes of a table have at least l-distinct values for the sensitive attribute. For the case of multiple sensitive attributes, one sensitive attribute is treated as the sole sensitive attribute, while the others are treated as quasi-identifiers. Thus, l-diversity sets requirements on both the quasi-identifiers and the sensitive attributes.

### Recommendations From Relevant Institutions

In this subsection, we examine privacy documents from public or independent bodies. We focus on recommendations or guidelines with respect to the usage, anonymization, and release of private or confidential data. Recommendations that are not applicable to research design, within the context of a research group or institution, and are specific to the public or independent bodies who issued the documents were filtered out. The recommendations are shown in Table 2 (some of those may have been paraphrased from the original reports) by each body, and divided into four categories according to the topic they address. The top part of the table shows the recommendations regarding the organization processes and training of the staff. The second category is about data processing, and the third category is about the publication of data and deliverables. The bottom part of the table shows recommendations regarding the release of data to a third body. Two public bodies provide recommendations with respect to confidential microdata (Centers for Disease Control and Prevention [CDC]-CSTE, 2005; Federal Committee on Statistical Methodology, 2005). Two bodies discuss social, health, or personal spatial data (Graham, 2012; Gutmann & Stern, 2007). Last, two bodies look into crime events as a special type of confidential discrete spatial data (Information Commissioner’s Office [ICO], 2012; Wartell & McEwen, 2001).

**Table 2.** Privacy and Confidentiality Recommendations From Public and Independent Bodies.

	FCSM	CDC-ATSDR		NRC	
Organization and training	1. Standardize and centralize agency review of disclosure-limited data products 2. Use consistent practices	1. Designate a privacy manager 2. Train all responsible staff 3. Define criteria for access to restricted-access files 4. Planning for release of PUDS		1. Methodological training in the acquisition and use of data 2. Training in ethical considerations of data that include explicit location information on participants 3. Design studies in ways that provide confidentiality protection for human participants	
	FCSM	CDC-ATSDR		ICO (POA)	
Data processing	3. Remove direct identifiers and limit other identifying information	5. Classify each dataset as a restricted-access or a PUDS		1. Increase a mapping area to cover more properties or occupants	
	FCSM	CDC-ATSDR	ICO (POA)	ICO (GCD)	NIJ–CMRC
Publication of data and deliverables	4. Share information on assessing disclosure risk	6. Include disclosure statement with PUDS 7. Maintain log of datasets rereleased	2. Reduce the frequency or timeliness of publication 3. Use mapping formats that do not allow the inference of detailed information 4. Avoid the publication of spatial information on a household level	1. The use of heat maps, blocks, and zones reduces privacy risks 2. New ways of representing information about crime should be explored	1. Decide which data to present: Point versus aggregate data 2. Use disclaimers to avoid liability from misuse or misinterpretation of data 3. Provide information on laws, liability, freedom of information, and privacy 4. Provide contact information of persons with privacy expertise and familiarity with the data
	CDC-ATSDR		NRC	NIJ–CMRC	
Release data to a third party	8. Authenticate the identity of data requestors 9. All restricted-access data requestors are required to sign a DSA 10. Requirements for a standard DSA for restricted-access data 11. Monitor user compliance with DSAs 12. Include addendum to the DSA when a requestor plans to link restricted-access data to other data 13. Include addendum to the DSA when a requestor plans further data releases from restricted-access data to other parties		4. Data stewards should develop licensing agreements to provide increased access to linked social-spatial datasets that include confidential information	5. Consider privacy and other implications if data provided will be merged with other data 6. Decide presentation of research results 7. Researchers and the agency decide what data will be needed 8. A nondisclosure agreement may be used to guarantee confidentiality 9. The agency can review any research results before publication 10. Perform background checks on research personnel who will have access to data 11. Decide where data will be stored to ensure secure settings 12. Require researchers to destroy raw data after the research is completed	

*Note.* Recommendations have been grouped into four categories according to the topic they address. FCSM = Federal Committee on Statistical Methodology; CDC-ATSDR = Centers for Disease Control and Prevention and the Agency for Toxic Substances and Disease Registry; PUDS = public-use dataset; ICO = Information Commissioner’s Office; POA = Practice on Anonymization; GCD = Geospatial crime data; NRC = National Research Council; NIJ = National Institute of Justice; CMRC = Crime Mapping Research Center; DSA = disclosure sharing agreement.

The U.S.-based Federal Committee on Statistical Methodology (FCSM) provides assistance and guidance on issues that affect federal statistics such as in situations when the Office of Management and Budget applies policies related to statistics. The most recent working paper on disclosure by the agency from 2005 discusses anonymization methods, practices employed by federal agencies, and offers recommendations for good practice for both tables and microdata. Another list of guidelines was published in a comprehensive report in 2005 by the Centers for Disease Control and Prevention and the Agency for Toxic Substances and Disease Registry (CDC-ATSDR). CDC and ATSDR are both U.S. federal agencies under the Department of Health and Human Services and therefore the focus of the report is on health data.

The recommendations by the National Research Council (NRC) in the United States and the independent body Information Commissioner’s Office (ICO) in the United Kingdom are specific to spatial confidential data. NRC provides services via reports to the government, the public, and the scientific or engineering communities. The recommendations address data collected by federal agencies, individual researchers, academic or research organizations, and outline the need to anonymize discrete spatial data. The code of practice on anonymization by ICO (named as ICO [POA] in Table 2) focuses on the requirements set by the Data Protection Act (The Stationery Office, 1998) to highlight key issues in the anonymization of personal data, and has a dedicated section on spatial information. Furthermore, ICO has published a separate report (named as ICO [GCD] in Table 2) with a focus on geospatial crime data. Due to the sensitivity of crime events and the increase of online crime mapping, the National Institute of Justice (NIJ) in the United States published as well a detailed report tailored to this topic. It discusses, among other issues, the publication of data and maps, and the sharing of data with other agencies or researchers.

Recommendations 1 and 2 from FCSM, 1 to 4 from CDC-ATSDR, and 1 to 3 from NRC suggest practices prior to the anonymization, release, or sharing of the data such as to offer essential training, establish a privacy plan, and standardize practices. There are a few recommendations regarding the processing of the data (3 from FCSM, 5 from CDC-ATSDR, and 1 from ICO [POA]), but they do not propose concrete anonymization methods. However, there are more precise recommendations when it comes to presenting spatial research outputs (2-4 from ICO [POA]; 1-2 from ICO [GPD], and 1 from NIJ). It is also recommended that a research output or a disclosed dataset is accompanied by privacy-related information (e.g., disclosure assessment, laws, liability, etc.) and a reference to contact person (4 from FCSM, 6 from CDC-ATSDR, 3-4 from NIJ). In addition, CDC-ATSDR suggests to maintain an inventory of released datasets. The inventory of restricted-access data should be stored internally to ensure compliance with the terms of the disclosure sharing agreement (DSA). On the contrary, for an anonymized public-use dataset (PUDS) the inventory can inform interested parties on the datasets’ availability and meta-data. Last, NIJ suggests the use of disclaimers to reduce liability when outputs, such as maps, may lead to ambiguous interpretations.

Regarding data releases to a third party (last category of Table 2), the bodies agree to the requirement of a formal agreement between the controller and the requestor. Also, checks of the requestor’s validity may be conducted (8 from CDC-ATSDR and 10 from NIJ). Then, the particulars of the data release and potential uses should be discussed and decided between the two parties such as merging released data with other data or presentation of results (12, 13 CDC-ATSDR and 5, 6, 7, 11 NIJ). Although data sharing particulars are decided with the DSA, the collector should still be allowed to review research outputs if needed.

### Privacy by Design Research Campaign

While previous research has mainly focused on methods to preserve privacy and measures to examine information disclosure, we propose practical privacy-preserving steps for the collection, storage, analysis, and dissemination of individual measurements from mobile participatory sensing applications. A privacy-preserving research campaign requires a concrete privacy plan of several tasks to be developed before, during, and after the completion of the campaign. These tasks are presented here as recommendations, because their application depends and varies based on a project’s specifications. In this article, we treat initial tasks as prior to starting a survey (subsection Presurvey Activities), storing, anonymization, and assessment of derived datasets (subsection Processing and Analyzing Collected Data), and actions to eliminate disclosure from published data and deliverables, or when datasets are shared with third parties (subsection Disclosure Prevention). Furthermore, a separate subsection is dedicated to recommendations that aim to ensure the appropriateness of the research environment to handle a privacy-preserving research campaign (subsection Security and Safety). In each subsection, we analyze and explicate the details of the recommendations which are then summed up on a table at the end of the respective subsections (Tables 3, 4, 5, and 7).

**Table 3.** A List of Initial Activities Prior to the Starting of the Survey.

A. Presurvey activities
1. Design study in the least privacy invasive manner
2. Develop a privacy-preserving research plan
3. Define criteria for access to restricted-access datasets
4. Prepare a participation agreement
5. Ensure inform consent on location privacy disclosure risks
6. Obtain institutional approval preferably reviewed from a DRB

*Note.* DRB = disclosure review board.

**Table 4.** A List of Recommendations to Ensure Secure and Safe Settings.

B. Security and safety
1. Assign a privacy manager
2. Train collectors and/or processors in methods and ethical considerations
3. Ensure a secure IT system
4. Ensure secure sensing devices

*Note*. IT = information technology.

**Table 5.** A List of Recommendations to Store, Anonymize, and Asses Derived Datasets.

C. Processing and analysis of collected data
1. Delete data from sensor devices once stored in the IT system
2. Remove identifiers from the dataset
3. standardize anonymization practices
4. Ensure that the inclusion of pseudonyms does not lead to disclosure
5. Ensure that the inclusion of quasi-identifiers does not lead to disclosure
6. Ensure a sufficient l-diversity of the sensitive attributes
7. Classify each dataset as a restricted-access or anonymized dataset
8. Assess disclosure of anonymized datasets
9. Assess anonymization effect on spatial analysis

*Note.* IT = information technology.

### Presurvey Activities

The privacy manager should initially design the study in the least privacy invasive manner depending on the purposes of the research study. For example, if analysis by user or group of users is not foreseen, all measurements can be stored altogether without pseudonyms. The study design should be reported within a research plan that has dedicated sections regarding privacy preservation. These sections should describe methods and practices that take place during the project’s duration, and for the time period for which personal data are to be kept by the team. Also if data are to be shared with third parties, criteria for access to restricted-access datasets (e.g., research personnel, data requestors) have to be defined and included in the plan.

The next presurvey step is the preparation of the participation agreement. Essential elements of a participation agreement include (a) purpose and procedures of the study, (b) potential risks and discomforts, (c) anticipated benefits, (d) alternatives to participation, (e) confidentiality statement, (f) injury statement, (g) contact information, and (h) voluntary participation and withdrawal (Hall, 2016). The confidentiality statement can vary depending on the location of the study area, and respective laws and regulations.

The participation agreement should outline the location privacy protection insertions in each stage of the project and communicate the remaining disclosure risks, if any. Those who communicate the study to the participants should explain in common language what is “location privacy” and other related terminologies, and provide examples that allow them to make an informed decision about whether to participate or not. An optional step for improvement in future surveys is to add the participants’ feedback regarding the perception and preferences on the established privacy measures.

Last, both the research plan and the participation agreement should go through institutional approval from objective and experienced staff of the institution or University such as IRB, review ethics committee (REC), or a more specialized disclosure review board (DRB). With respect to the type of organization, De Wolf (2003) suggests to consult a cross-disciplinary DRB that makes recommendations to the IRB, if the institution’s IRB does not have a standardized process for reviewing outputs from survey confidential data. The creation of a cross-disciplinary DRB could also serve as a committee that educates researchers on the current available anonymization and disclosure techniques.

### Security and Safety

The first step of a research campaign that collects participatory sensing data is to assign a dedicated privacy manager who is responsible for the tasks of this subsection as well as for consulting on (or performing) the tasks of the following subsections. The privacy manager should train data processors and collectors regarding their specific activities, and is also responsible to ensure that the research environment provides secure and safe settings regarding the sensing devices and the information technology (IT) system where data will be stored and processed.

With regard to the security of IT systems, Boulos et al. (2009) provide a comprehensive list of measures that include the usage of (a) advanced cryptography, (b) biometrics, (c) unlocking the data under the physical presentation of other members, (d) cable locks, (e) computers with a built-in trusted platform module (TPM) chip, (f) password attack protection, (g) network security, (h) multilevel security (MLS), (i) secure USB flash drives, (j) blanking computer display and autolog-off, (k) discarding of old equipment and storage media.

Furthermore, security should be scrutinized on the sensing devices. Tracking subjective observations is typically performed via smartphone “human-as-sensor” applications that are developed by research teams tailored to the requirements of a research study (Solymosi et al., 2015; Zeile, Resch, Loidl, Petutschnig, & Dörrzapf, 2016). It is recommended that the application does not incorporate a closed-source third-party code. In this case, the researchers cannot accurately estimate the risk because they cannot be certain that the third party will not appropriate the sensed data. Instead, the “human-as-sensor” software should be developed exclusively by the research team. Also, data should be stored only locally and in an encrypting form to prevent the security risks during transmission, when data are stored in the cloud, and when devices are lost or stolen. Collected data should be transferred regularly to the secure research IT system.

Also, objective observations are tracked with products (smartphone applications or wearable devices) that measure physiological measurements. Although a research campaign may develop and use their own product (Bergner, Zeile, Papastefanou, & Rech, 2011; Zeile, Höffken, & Papastefanou, 2009), professional products may be purchased as well from specialized sensor companies. This means that researchers analyze collected data (outputs) of “blackbox” systems. When these systems operate on smartphones that may have access to other applications and sensors of the device data security risks are harder to estimate. Thus, we recommend the purchase and use of wearable devices. Similar to the “human-as-sensor” applications, data should be stored only locally and in an encrypted form.

In addition, Bluetooth and Wi-Fi should be turned off while the participants use the devices. If this is not possible and the survey is conducted for longer periods of time, the devices should be randomly and regularly interchanged among the participants. Therefore, if the trajectories of a device are collected by a scanner, they could not be linked to a single individual. The research group may empty the devices and store the data before each exchange (e.g., on a daily basis) to retain the trajectories of each participant distinguishable.

If a research team opts for third-party smartphone applications (for collecting either subjective or objective sensing measurements) which transmit and store data on the cloud, the relevant security risks have to be considered and communicated to the participants of a survey.

### Processing and Analyzing Collected Data

The processor should empty sensor devices once data have been archived, and remove identifiers from the dataset. According to the Health Insurance Portability and Accountability Act (HIPAA) Privacy Rule, there are 18 elements that should be either removed or generalized to deidentify a dataset (U.S. Government Publishing Office, 2009). These are (a) names; (b) geographic subdivisions smaller than a State with some exceptions having a population threshold of 20,000 people; (c) dates directly related to an individual; (d) telephone numbers; (e) fax numbers; (f) electronic mail addresses; (g) social security numbers; (h) medical record numbers; (i) health plan beneficiary numbers; (j) account numbers; (k) certificate/license numbers; (l) vehicle identifiers and serial numbers, including license plate numbers; (m) device identifiers and serial numbers; (n) Web Universal Resource Locators (URLs); (o) Internet Protocol (IP) addresses; (p) biometric identifiers, including finger and voice prints; (q) full-face photographic images and any comparable images; and (r) any other unique identifying number, characteristic, or code. If necessary, identifiers linked to pseudonyms or measurements may be kept in a separate encrypted database to allow original data and study results to be sent to the participants. Also, the deletion of data and removal of identifiers may be a daily task or a regular task during the survey when it is conducted for longer periods of time.

The next step is data anonymization. The anonymization of an identifier’s free spatial dataset is necessary as long as data subjects are to be distinguished from each other. If multiple datasets are to be collected by the research campaign, the anonymization approach should be standardized to ensure consistency on released datasets. Collected data should be anonymized prior to their release considering the following three principles: (a) inclusion of pseudonyms does not lead to disclosure, (b) inclusion of quasi-identifiers does not lead to disclosure, and (c) sensitive attributes are “well represented” among the equivalent classes of quasi-identifiers. All processed datasets should be classified as restricted-access and anonymized datasets.

An inevitable result of the anonymization process is the reduced quality and accuracy of the anonymized dataset. In fact, by increasing the privacy levels of an anonymized dataset the dissimilarity of the dataset to the original one will also increase. Nevertheless, the analytic usefulness also depends on the anonymized method. For example, anonymized data based on the donut method, random perturbation, and adaptive areal elimination performed better in detecting spatial clusters compared with aggregation for the same level of spatial k-anonymity (Hampton et al., 2010; Kounadi & Leitner, 2016). Hence, the person who is responsible to anonymize should select the approach that has the least effect on the analysis to be performed by future data users, conditioned that the approaches can offer the same level of anonymity.

For example, if the relationship between the locations of measurements and other covariates is important, the synthetic geographies may be an ideal approach. For clustering and pattern analysis, we suggest adaptive geomasking, dummies, or mix zones. While geomasking retains the count of the original dataset, dummies add data, and mix zones remove data from the dataset. Hence, they should be preferred in highly populated areas of low sensitivity where it is more likely that the addition or removal of measurements has a minimal effect. If data are to be used for areal analysis or for choropleth mapping, cloaking can be used as a form of adaptive areal aggregation. The data will be less precise than the original data; however, there will be no spatial error involved. On the contrary, the usefulness of the cloaked areas should be considered because they may vary in size and also overlap other analysis units such as administrative areas. In such scenarios, areal interpolation can be performed that also involves a spatial error to be estimated. Also, point aggregation, as a form of generalization, can be used to visualize the measurements’ trajectories. Again, there is no spatial error but less precise data.

The final step is the assessment of the anonymized data regarding the disclosure risk, if any, and the anonymization effect of the quality of the masked data. The assessment should be clearly communicated to potential users. In Table 6, we present measures that can be used to quantify the effect of the anonymization process to the masked data based on the type of spatial analysis to be performed. The global divergence index (GDi) is a composite indicator which considers the spatial mean as a measure of central tendency, the orientation of the ellipse as a measure of directional trend, and the length of the ellipse’s major axis as a measure of spatial dispersion (Kounadi & Leitner, 2015). It shows the divergence of global spatial statistics of the masked point pattern to the original point pattern. For point pattern analysis and detection, possible approaches are to calculate *Cross K function* analysis (Kwan et al., 2004), *distance to k-nearest neighbor* (Seidl et al., 2015), or *Moran’s I value* to both masked and original datasets, and report the differences of the results. When locations of masked events are used in univariate spatial prediction, the prediction accuracy index (PAI; Chainey, Tompson, & Uhlig, 2008) and the prediction efficiency index (PEI; Hunt, 2016) can be used to evaluate the predicted hotspot areas where the events are more likely to occur. Then, the PAI and PEI of masked and original datasets can be compared and reported.

**Table 6.** Measures to Evaluate the Anonymization Effect by Type of Spatial Analysis.

Unit of analysis	Spatial analysis	Measures of spatial error and information loss
*Points*	Global descriptive statistics	Global divergence index (GDi)
	Pattern detection/analysis	Divergence to clustering distance in cross *K* function analysis, distance to *k*-nearest neighbors, or Moran’s *I* value
	Univariate spatial prediction	Divergence to prediction accuracy index (PAI), prediction efficiency index (PEI)
	Local indicators of spatial association	Local divergence index (LDi), stability of hotspot (SoH)
	Spatial clustering	Detection rate, accuracy, sensitivity, and specificity
	Multivariate spatial relationship	Divergence to *R*-squared or root-mean-square standardized error
*Areas*	Choropleth mapping, density surface estimation	Index of similarity (S), suppression, compactness, discernibility, nonuniform entropy

The local divergence index (LDi) calculates the divergence of hotspot areas using the Getis-Ord Gi* statistic. This index can be used to detect the masking effects to the local characteristics of the original pattern. Another approach that can be used for the local properties is the Stability of Hotspot (SoH) metric which was originally designed to measure the clusters’ deviation from the same datasets in different resolutions (Bruns & Simko, 2017). The same metrics can be used to measure the clusters’ deviation from different datasets (original vs. masked) of the same resolution. Regarding spatial clustering, there are a few indices that can be used. *Clusters’ detection rate* is the percentage of significant spatial clusters (Olson, Grannis, & Mandl, 2006), *clusters’ accuracy* is the percentage of significant clusters in which at least half of the masked points originate from clustered original points (Olson et al., 2006), clusters’ sensitivity is the percentage of masked points that originate from clustered original points and are still clustered (Cassa et al., 2006; Hampton et al., 2010), and clusters’ specificity is the percentage of masked points that originate from nonclustered points and are still nonclustered (Cassa et al., 2006; Hampton et al., 2010). Regression models such as geographically weighted regression (GWR) or spatial regression can be applied to the original data and a covariate(s) (explanatory variable), and then to the masked data and the covariate(s). The divergence of the models’ results, such as *R*-squared or root-mean-square standardized error, can act as a measure of error in prospective multivariate analysis.

Regarding analysis on areas or grid cells, the index of similarity *S* can identify the degree to which counts within areal units is different (Andresen, 2009; Tompson, Johnson, Ashby, Perkins, & Edwards, 2015). Furthermore, aggregation-based anonymization techniques are ideal for choropleth mapping or density surface estimation. Aggregation does not affect the accuracy but the precision of the data. Therefore, the effect can be evaluated with information loss metrics such as suppression (i.e., number of suppressed records), compactness (indicates level of geographic precision), discernibility (checks for anonymity levels higher than the desired level), and nonuniform entropy (based on the probability of identifying original locations; Croft, Shi, Sack, & Corriveau, 2017).

Measures that are in the form of an index or a standardized metric should be preferred because they allow comparisons between datasets and study areas that are not possible for some of the measures listed in Table 6. For example, it may be useful to calculate the divergence of masked and original data to the third nearest neighbor distance by three anonymization approaches, and identify the approach that has the least effect on point pattern analysis. However, this measure cannot be used to compare the effects of two datasets in different areas that were anonymized in the same way. In this scenario, the divergence to Moran’s *I* values or to another global statistic of spatial autocorrelation with fixed intervals can be employed. The use of indices and standardized metrics allows the testing with several datasets and areas, and can give an overall evaluation of anonymization technique for its usage in spatial analysis.

### Disclosure Prevention

Dissemination of research findings poses significant privacy threats as those discussed in Disclosure Scenario 5 (Subsection Disclosure Risk of Data and Deliverables). Hence, researchers should carefully evaluate their research outputs and only present findings, particularly in the form of a map, if these are needed to convey important messages to the readers of a publication. A simple way to avoid disclosure risks is to decrease the spatial and/or the temporal precision of findings. While researchers may want to report on details of the study area and collected data, they should avoid point distribution maps of original data in cases where participants can be distinguished (e.g., different coloring per participant or groups of participants, or each point indicates a private location about one participant). Haley et al. (2016) did a literature review on articles published in PubMed, and identified numerous cases that displayed participant data in maps as points or small-population geographic units. In more than half of the articles, the authors either did not refer to employed privacy protection approaches or anonymized data inadequately. Safe alternatives to point distributions can be a density surface estimation or a clustering spatial distribution that reduce the risk of spatial reidentification. However, these practices may portray a negative or positive image about an entire neighborhood that will be perceived as a hotspot of the sensed measurement.

If it is necessary for research purposes to present a sensitive point map, anonymization techniques such as these under the category “confidential discrete spatial data” of Table 1 should be employed. However, the masked point distribution will, to some degree, be different from the point distribution of the original dataset. The researchers should consider this error and the impact it may have on reader’s interpretation of the map. Also, it is important to mention that location privacy risks appear when participatory data are collected for longer periods of time for which the participant has the device on, meaning that his or her identifying locations can be captured (for more details, refer to inferences on places under Disclosure Scenario 1, Subsection Disclosure Risk of Released Data and Deliverables). Hence, a participant distinguishable map poses no privacy risks if data are collected for a clearly defined study area or route, and no further identifying information about the participants is included on the map. If there are any disclosure risks associated with a published map, the responsible researcher must estimate and report them. Last, when research outputs are uploaded on a research project’s web page the usage of disclaimers may limit unintended misconceptions of the presented information. There are no standard disclaimers for use, but they depend on the publication and information prone to interpretation. The wording should specify what does the publication is not liable for, such as decisions and actions taken by a reader, and errors in the data such as omissions, systematic bias, or inaccuracies due to privacy constraints.

Furthermore, anonymized datasets may be disclosed as long as data are protected and follow the recommendations below. There are different reasons behind an institution’s or research group’s decision to share their data. A research group may wish to make their collected data publicly available to increase visibility of their work, and allow other researchers to use them which will in turn make scientific comparisons possible. On the contrary, releasing data may be a compromise against the will to publish in a scientific journal that has a data policy which requires research data to be publicly available (PLOS ONE, 2014). In such cases, a document should be attached to the released datasets that contains information on the disclosure risk, protection method, masked data quality, and contact information on privacy matters. In addition, practices that increase the disclosure risk such as the release of multiple versions of anonymized datasets or disclosure of anonymization meta-data should be avoided.

It is also possible that collected spatiotemporal participatory data are shared with other institutions or researchers. Data sharing should be one of the many privacy insertions of the confidentiality statement within the participation agreement. The institution is responsible for preparing a licensing agreement for such purposes regardless of the data nature (i.e., anonymized data or restricted-access data). For restricted-access data, a separate DSA should be prepared, or a respective section within the licensing agreement should be inserted. Recommendations 17 to 23 of Table 7 are intended mainly for restricted-access data. It is advisable that the institution performs checks on the credibility and capability of the requestor to handle sensitive personal data such as investigating the requestor’s research personnel, settings, and identity. The controller and the requestor should decide together about the data that are needed, the length of period that will be kept by the requestor, and examine potential linkage-disclosure implications if original data are to be used with other datasets. Regarding research outputs, the controller should have the right to review the presentation as well as the final publication deliverables to ensure that anonymity is preserved. Last but not least, the privacy manager should maintain an inventory of all disclosed or shared datasets that describes the datatype based on the classification, the disclosed destination (e.g., another institution, open data platform), and other relevant information.

**Table 7.** A List of Recommendations to Prevent Disclosure When (a) Findings Are Published, (b) Anonymized Datasets Are Published, and (c) Data Are Shared With Third Parties.

D. Disclosure prevention
Dissemination of findings
1. Reduce spatial precision
2. Reduce temporal precision
3. Consider alternatives to point distribution maps
4. Assess disclosure on a point distribution map
5. Provide protection vs. disclosure information
6. Provide contact information
7. Use disclaimers
Anonymized datasets
8. Avoid the release of multiple versions of anonymized datasets
9. Avoid the disclosure of anonymization meta-data
10. Inform about disclosure risk assessment
11. Provide information on protection and effect
12. Provide contact information
13. Maintain log of anonymized disclosed datasets
Data sharing with third parties
14. Plan a mandatory licensing agreement
15. Plan a DSA for restricted-access data
16. Authenticate the identity of data requestors
17. Perform background checks on research personnel who will have access to data
18. Ensure requestor’s safe settings
19. Decide what data will be needed
20. Consider implications if restricted-access data will be merged with other data
21. Decide presentation of research outputs
22. Decide length of period of retaining restricted-access data
23. Review research outputs before publication
24. Maintain log of restricted-access disclosed datasets

*Note*. DSA = disclosure sharing agreement.

## Conclusion

The proposed privacy recommendations were generated from two sources of information: The first source of information is the technical information, and second one is the experts’ suggestions. Technical information includes the disclosure risk and approaches to minimize or eliminate the risk. The experts’ suggestions are a summary of recommendations or guidelines regarding confidentiality issues that arise from the collection, use, or dissemination of personal data. A chronological classification of our recommendations involves first these that should take place before the initiation of the survey (presurvey), second these that ensure the safety and security of the research environment, next as soon as data are collected (processing), and finally after data are processed. Some recommendations are applicable to all research projects (e.g., ensuring safe settings or the privacy protection of research outputs). However, recommendations regarding the disclosure of anonymized datasets and sharing restricted-access data with third parties are applicable only if the data controller opts for these practices. Our set of recommendations can act as a general guideline for research campaigns that want to use participatory sensing data by enlisting the steps of the campaign where privacy actions should be taken. Some of our recommendations, such as anonymization and dissemination of findings, can also be applicable to other types of spatial data. However, privacy restrictions that may be specific to other types of data and the bodies that share them are not discussed here.

An important prerequisite of any research project that involves spatiotemporal participatory data is that the members of the project are either trained or experts in location privacy threats. The training should take place at an early stage of the research campaign to guarantee success in the next two tasks: The first task is to prepare the research plan and the participation agreement. If the data collector decides to share sensitive data with third parties, criteria for sharing restricted-access data (i.e., identifier-free survey data) should be included in the research plan. Both the research plan and the participation agreement should be comprehensive regarding the privacy insertions to ensure a successful institutional approval of the survey. The second task is to ensure that the research environment establishes secure measures to prevent privacy and confidentiality breaches of collected and stored data.

The processing tasks start as soon as survey data are collected. First, data should be safely stored, the devices should be cleaned from any stored data, and identifiers should be removed from the datasets to be analyzed. These basic yet critical steps are frequently neglected during the processing of survey data. The removal of direct identifiers is a prerequisite to deidentify the data, but if quasi-identifiers and pseudonyms are to be included an anonymization approach should be employed as well. As a general principle, the analysis to be performed should be the guide for selecting an anonymization technique to minimize the effect of masked data to the accuracy of spatial analysis (e.g., clustering, point pattern, multivariate, etc.).

Then, the research team should calculate the anonymization effect of the masked data on spatial analysis, evaluate the remaining disclosure risk, and classify all stored datasets as “anonymised” or “restricted-access” datasets. Regarding the anonymization effect, we suggested measures to evaluate the error or information loss of the masked data in spatial analyses. We focused on measures that quantify the magnitude of the effect and, whenever possible, have been used in the geoprivacy literature because their usage in future studies would allow comparison of results.

The last set of recommendations refer to the tasks after data are processed. First, the members of the research campaign should examine the disclosure risk of their research outputs, such as maps in scientific journals, and apply a protection approach if private locations of measurements are to be published. Second, to ensure ethical conduct of research we suggest reporting generally on the employed privacy protection practices of outputs or anonymized data as well as adding disclaimers. Third, careful consideration should be taken while releasing and reporting on anonymized datasets so as not to provide disclosure hints to a potential privacy attacker. Fourth, the privacy manager should prepare licensing and DSAs, and maintain a data inventory of all published or shared datasets. Last, the controller must investigate the appropriateness of the requestor’s environment and personnel to handle sensitive data, and he or she should have an active role regarding the privacy-preservation practices of the requestor’s research plan.

This set of recommendations establishes ethical scientific practices and ensures sufficient privacy protection which are crucial elements so as to engage people to contribute actively being “human data sources.” This is necessary to leverage collective information in areas such as environmental monitoring, urban planning, security and quality of life, emergency management, traffic monitoring, or e-tourism. Nonetheless, the willingness to voluntarily share personal data is linked with the trust in the security of the data. To make an informed decision on the data’s security, participants need to be aware of the potential misuses, countermeasures, and their efficiency. Yet, privacy-related terms, conditions, and technology are mostly hardly understandable to nonexperts. Therefore, more simple and binding ways of communicating this kind of information have to be found.

## Best Practices

Best practices are discussed in detail in the recommendations sections: Presurvey Activities, Security and Safety, Processing and Analyzing Collected Data, and Disclosure Prevention. The most critical practices are summarized in the “Conclusion” section.

## Research Agenda

Anonymization and disclosure risk evaluation are important tasks of a privacy-preserving research campaign that require further empirical research. Regarding the anonymization, we emphasized on approaches that are heavily discussed in the geoprivacy literature, but we do not claim that this is a comprehensive list. Additional methods should be explored, especially in situations when the anonymization needs to be tailored to the specifications of a research campaign and collected survey data. We discussed how the qualities of participatory sensing data urge for a fusion of anonymization methods that consider both k-anonymity and l-diversity. Currently, there is a lack of methods specified to these qualities that can successfully prevent all types of disclosure.

Also, there has been limited discussion on the evaluation or quantification of the disclosure risk. Some of the scholars who developed anonymization methods have either quantified the disclosure risk with formulas that are typically specified to their method, or developed a method conditioned that it preserves an estimated anonymity (Allshouse et al., 2010; Beresford & Stajano, 2004; Croft et al., 2016; Paiva et al., 2014; Wieland, Cassa, Mandl, & Berger, 2008; You et al., 2007; Zhang, Freundschuh, Lenzer, & Zandbergen, 2017). However, the results or conclusions of these studies should not be generalized because the characteristics of a study area or available linked datasets and background information of the original dataset can vary. Therefore, a different approach to evaluate the disclosure risk may be needed. Furthermore, not all anonymization methods were assessed regarding the disclosure risk they entail. However, there are some studies who looked at the disclosure risk of original data. For instance, Alrayes and Abdelmoty (2014) examined aspects of potential personal information that may be derived from LBSN data. The estimated potential problems are not verified by means of actual disclosure due to the fact that LBSN validation data are hard to obtain (e.g., real private locations or identity of users). De Montjoye, Hidalgo, Verleysen, and Blondel (2013) analyzed mobile phone data, and found that four randomly chosen points are enough to uniquely characterize 95% of heavy users drawn from a random sample. Nevertheless, the extent to which these four locations can lead to a successful inferential or attribute disclosure of the users’ personal information (e.g., identity or household location of a user) remains unexplored. Thus, the evaluation of the disclosure risk is still a topic that needs to be examined in depth with empirical studies that involve validation data.

## Educational Implications

Current research in location privacy has revealed that researchers that use spatial data do not always employ adequate privacy-preserving practices. This can be partially attributed to the lack of scientific expertise and technological background. To eliminate future practices that may compromise individual privacy, every research campaign that collects participatory sensing data should assign a privacy manager. The privacy manager must be trained in the following areas:

- Anonymization techniques and location protection methods
- Estimation of the disclosure risk
- Analytical methods of participatory sensing data
